# Anti-MDA5 juvenile idiopathic inflammatory myopathy with second-degree heart block but no skin or lung involvement: a case report

**DOI:** 10.1186/s41927-021-00180-9

**Published:** 2021-04-02

**Authors:** Meghan E. Ryan, Daniel Cortez, Kelly R. Dietz, Peter Karachunski, Bryce A. Binstadt

**Affiliations:** 1 Department of Pediatrics, Division of Rheumatology, Allergy & Immunology, University of Minnesota, Minneapolis, USA; 2 Department of Pediatrics, Division of Cardiology, University of Minnesota, Minneapolis, USA; 3 Department of Radiology, University of Minnesota, Minneapolis, USA; 4 Department of Neurology, University of Minnesota, Minneapolis, USA

**Keywords:** Case report, Juvenile idiopathic inflammatory myopathy, Heart block, Anti-melanoma differentiation-associated protein 5

## Abstract

**Background:**

Patients with idiopathic inflammatory myopathy and autoantibodies directed against melanoma differentiation-associated protein 5 (MDA5) characteristically have interstitial lung disease, severe cutaneous involvement, arthritis, and relatively mild myositis. Cardiac involvement in idiopathic inflammatory myopathy can occur and has been associated with anti-signal recognition particle and anti-polymyositis-scleroderma autoantibodies, but not with anti-MDA5 autoantibodies.

**Case presentation:**

A 14-year-old male presented with weakness, second-degree heart block, arthritis, and hematologic cytopenias. Imaging and biopsies confirmed the diagnosis of juvenile idiopathic inflammatory myopathy, and he had high titer anti-MDA5 autoantibodies. There were no cutaneous or pulmonary abnormalities. While on prednisone and methotrexate, the patient’s heart block improved from second- to first-degree and the cytopenias resolved. Persistent myositis prompted the addition of intravenous immunoglobulin. Seven months into the disease course, the arthritis and myositis are in remission and the patient is no longer taking corticosteroids.

**Conclusions:**

We report a novel case of a patient with juvenile idiopathic myositis who lacked the typical cutaneous and pulmonary findings associated with anti-MDA5 positivity, but who had cardiac conduction defects. This report broadens the clinical spectrum of anti-MDA5-associated inflammatory myopathy.

## Background

Patients with idiopathic inflammatory myopathy (IIM) and autoantibodies directed against melanoma differentiation-associated protein 5 (anti-MDA5) characteristically have interstitial lung disease (ILD), severe cutaneous involvement, arthritis, and relatively mild myositis. Arthritis is reported in 100% of IIM patients with anti-MDA5 antibodies. The other features vary in incidence between study populations but appear to occur more commonly in patients with anti-MDA5 compared to other myositis autoantibodies [1–5]. Cardiac involvement and the lack of either cutaneous or lung abnormalities in juvenile IIM (JIIM) patients with anti-MDA5 antibodies has not been documented previously. We describe a unique case of a patient with anti-MDA5-associated JIIM.

## Case presentation

A 14-year-old male with history of alopecia areata presented with a 2-month history of progressive fatigue, 5 kg weight loss, muscle weakness, and painful swollen joints. Physical examination revealed arthritis in the wrists and several finger joints, myalgias with initially normal strength, and a small patch of alopecia on his head. He had no rash, and his lungs were clear. He underwent an extensive evaluation for malignancy, infections, and systemic rheumatic diseases. Evaluation was notable for cytopenias (nadir leukocytes 2.7 × 10^9^/L, lymphocytes 0.9 × 10^9^/L, hemoglobin 10.5 g/dL, platelets 137 × 10^9^/L), but examination of the bone marrow was normal. Anti-platelet antibodies were not detected. Parvovirus titers demonstrated past exposure. He had intermittently elevated muscle enzymes (AST 83 U/L [reference range 0–50 U/L], aldolase 10.9 U/L [reference range 3.3–9.7 U/L], CK 318 U/L [reference range 30–225 U/L], LDH 694 U/L [reference range 0–337 U/L]), and normal von Willebrand factor antigen at initial presentation.

**Fig. 1:**
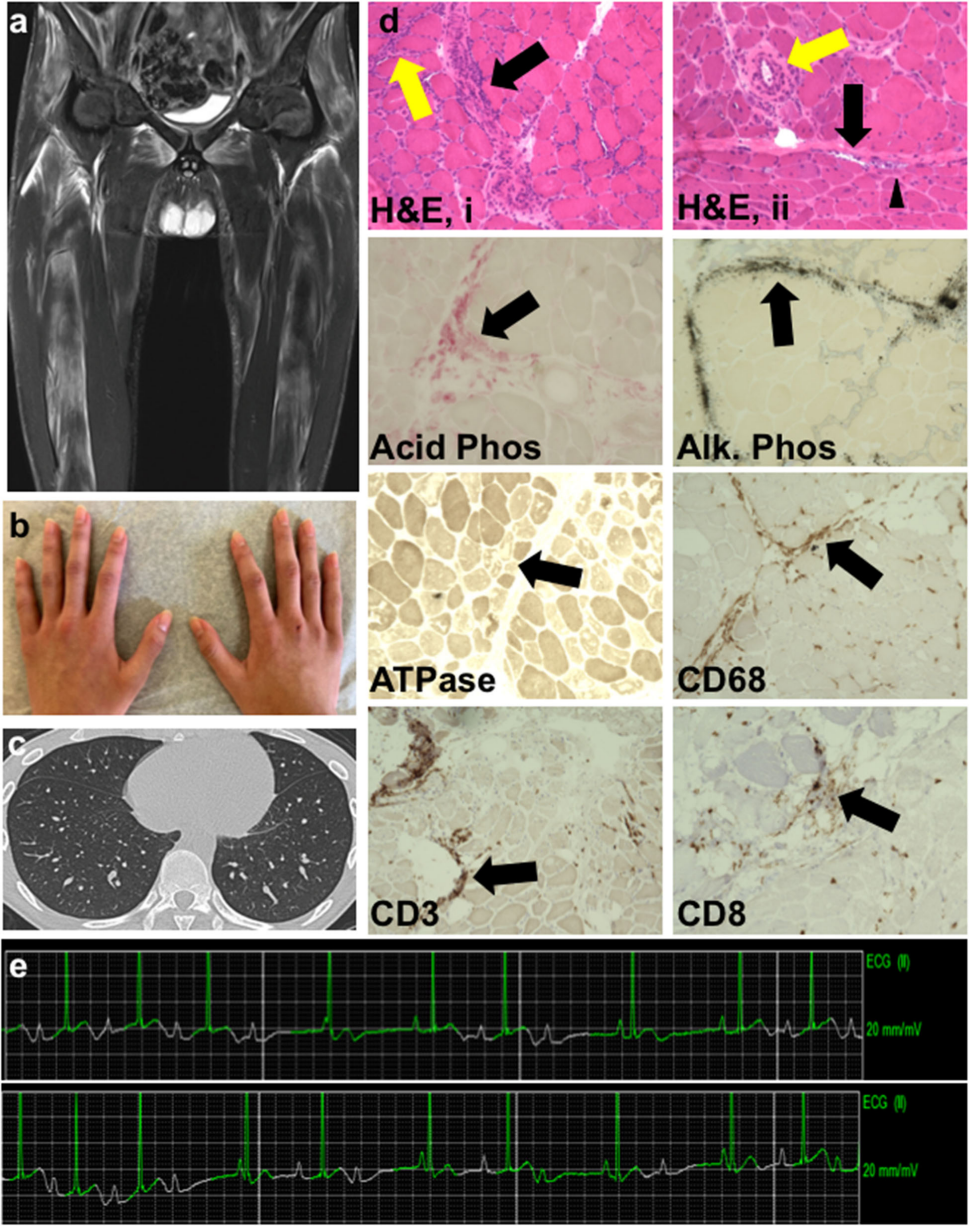
Representative images from an adolescent with anti-MDA5 JIIM. **a** Pelvis and thigh MRI without contrast coronal STIR view demonstrating patchy increased signal intensity. **b** Absence of rash on hands **c** Normal non-contrast chest computed tomography, axial view **d** Muscle biopsy stained with the indicated dyes or antibodies. H&E, i: Perimysial (black arrow) and endomysial (yellow arrow) inflammatory infiltrates. H&E, ii: Highlights marked fiber size variation with perifascicular atrophy with fibers showing purple discoloration and disrupted internal architecture (black arrow), perivascular inflammation (yellow arrow), many muscle fibers have central nuclei and vacuolar degeneration (arrowhead). Acid Phosphatase: Red staining predominantly in perimysium highlights inflammation involving histiocytes. Alkaline Phosphatase: Black staining highlights perimysial connective tissue reactivity. ATPase shows highlights of perifascicular atrophy and patchy loss of staining indicative of necrosis and degeneration (black arrow). Anti-CD68 staining localized to areas of inflammation involving histiocytes, especially in perimysium (arrows). Anti-CD3 and CD8 staining localized to areas of T-cell lymphocytic infiltrates (arrows). **e** Telemetry: top: Bradycardia with Wenckebach; bottom: 2:1 atrioventricular block

Magnetic resonance imaging (MRI) of his pelvis and proximal thighs revealed both myositis and fasciitis (Fig. 1a). Examination of his hands showed no cutaneous disease (Fig. 1b). Computed tomography of the chest was normal (Fig. 1c). A muscle biopsy from his left quadricep muscle demonstrated findings consistent with inflammatory myopathy similar to dermatomyositis (Fig. 1d). The patchy distribution of myositis found on MRI and muscle histology correlated with his mild myositis on exam. A myositis autoantibody panel revealed high positive anti-MDA5 antibodies but no other specific antibodies. Intermittent, asymptomatic high-grade second-degree heart block was identified via electrocardiogram and telemetry (Fig. 1e). Cardiac laboratory evaluation was notable for transiently elevated N-terminal pro b-type natriuretic peptide 820 pg/mL [reference range 0–240 pg/mL], but troponin I ES was normal. No additional cardiac testing was done.

He was diagnosed with JIIM and started on oral methotrexate (15 mg weekly) and oral prednisone (20 mg daily). After 2 weeks of treatment, he continued to have active myositis. Therefore, he was started on intravenous methylprednisolone (1 g weekly) in addition to increased oral methotrexate (25 mg weekly) and oral prednisone (40 mg daily). One month into therapy, he had mild improvement in his myalgias and improvement in his heart block from second- to first-degree. His cytopenias resolved and muscle enzymes improved but remained slightly elevated. His von Willibrand factor antigen was now mildly elevated. Pulmonary function testing was normal with DLCO of 72%. Monthly intravenous immunoglobulin (IVIG) was added due to persistent weakness and myalgias. His myositis improved more significantly with the addition of IVIG, allowing tapering and eventual discontinuation of the corticosteroids. Five months into treatment, the patient continues to lack the cutaneous or pulmonary features characteristically associated with anti-MDA5 seropositivity. His arthritis has resolved, strength is normal, and muscle enzymes have normalized. His most recent pulmonary function tests were again normal with an improved DLCO of 83%.

## Discussion and conclusions

There are no prior reports of patients with anti-MDA5 JIIM who lack both cutaneous and pulmonary features. In a recent case series that included 13 patients with anti-MDA5 JIIM, 9 patients had ILD at some point in their disease course. The onset of ILD occurred a median of 4 months after diagnosis with one patient developing ILD as late as 85 months after diagnosis [3]. Hence, it is possible that our patient could still develop pulmonary involvement.

This patient uniquely had second-degree heart block which has not been previously described in anti-MDA5 positive JIIM patients. In general, cardiac abnormalities are uncommon in patients with JIIM. A large, multicenter study reported cardiac abnormalities in only 2.9% of > 400 juvenile dermatomyositis patients, including myocarditis, pericarditis, and conduction defects; however, this study contained no information regarding autoantibodies [6]. Myositis autoantibodies previously associated with cardiac abnormalities in JIIM patients include anti-signal recognition particle and anti-polymyositis-scleroderma [5, 7]. To our knowledge, there is only one other case report of conduction abnormalities in anti-MDA5 IIM: a 68-year-old female who had complete heart block [8]. We speculate that our patient had inflammatory changes within the myocardium that resulted in the observed conduction abnormalities. The resolution of his heart block after immunomodulatory treatments is consistent with this notion. Literature regarding the responsiveness of IIM-associated conduction defects to immunomodulatory therapy is mixed, with one report demonstrating improvement in some children, but not in adults [9].

In conclusion, cardiac conduction abnormalities can occur in anti-MDA5-associated JIIM. Furthermore, JIIM can arise in the absence of ILD or cutaneous abnormalities. Close monitoring for ILD is recommended for IIM patients with anti-MDA5 seropositivity. Evaluation for cardiac conduction defects and other cardiac abnormalities is also recommended.

## Data Availability

Not applicable.

## Abbreviations

AST: Aspartate aminotransferase
CK: Creatine kinase
DLCO: Diffusing capacity for carbon monoxide
IIM: Idiopathic inflammatory myopathy
ILD: Interstitial lung disease
IVIG: Intravenous immunoglobulin
JIIM: Juvenile idiopathic inflammatory myopathy
LDH: Lactate dehydrogenase
MRI: Magnetic resonance imaging
MDA5: Melanoma differentiation-associated protein 5
